# Change in negative mental filter is associated with depression reduction in metacognitive training for depression in older adults (MCT-Silver)

**DOI:** 10.1038/s41598-024-67063-0

**Published:** 2024-07-25

**Authors:** Brooke C. Schneider, Ruth Veckenstedt, Evangelos Karamatskos, Jakob Scheunemann, Steffen Moritz, Lena Jelinek, Franziska Miegel

**Affiliations:** Department of Psychiatry and Psychotherapy, University Medical Center Hamburg-Eppendorf, Martinistr. 52, 20246 Hamburg, Germany

**Keywords:** Metacognition, Geriatric depression, Cognitive-behavioral therapy (CBT), Thought distortions, Session-specific effects, Affective disorders, Psychotherapy, Psychology, Depression

## Abstract

Identifying components of modularized psychological interventions that contribute to symptom reduction is essential to improving depression treatment. In a secondary analysis of a randomized controlled trial (RCT), session-specific effects of Metacognitive Training-Silver, a group intervention for older adults with depression, were investigated. Thirty-eight older adults with major depressive disorder or dysthymia participated in up to eight sessions of MCT-Silver. A clinical assessment of depressive symptoms (Hamilton Depression Rating Scale) as well as additional interviews and questionnaires administered as part of the RCT were completed at pre- and post-intervention. Depressive symptoms, negative (meta)cognitive beliefs, emotion regulation strategies and attitudes toward aging were assessed pre- and post-session. The rate of change in each variable per module, elevation following the module in which the variable was addressed, and the rate of change post module were examined via linear mixed models. Clinician-rated depressive symptoms were significantly reduced from pre- to post-intervention (Cohens *d* = 1.31). Self-reported depression and negative mental filter measured within sessions improved significantly over treatment, whereas black-and-white thinking improved after module #3 (Should Statements, All or Nothing Thinking and Acceptance). Module-specific within-session effects were found for overgeneralization (module #1: Mental Filter) and rumination (module #6: Rumination and Social Withdrawal). Improvement in mental filter in module #1 was significantly associated with depression reduction. This study provides initial evidence that MCT-Silver partially meets its aims of reducing depression and specific cognitive variables within and across sessions. Improvement of the instrument used to measure change may improve detection of module-specific effects.

Trial registration: NCT03691402.

## Introduction

Depression remains one of the most prevalent mental health conditions in later life with over 35% of older adults (ages 60 years and older) reporting clinically significant symptoms globally^1^. Guidelines recommend psychotherapy, medication, or both for the treatment of depression in older adults; however, anti-depressant use is often limited due to polypharmacy, adverse effects, and frailty. Instead, some guidelines, such as those in Germany and North America, indicate that older adults should be offered psychological interventions as a first-line approach^2,3^ and cognitive-behavioral (CBT) group interventions have garnered significant empirical support^4^. One major barrier to realization of these treatment guidelines is the large treatment gap, particularly for older adults seeking psychological treatment. In studies across Europe, only 11–21% of older adults meeting criteria for mental health disorders reported receiving services^5,6^. To help close this mental health care gap and to bolster healthcare systems for the increase in mental health care needs for an aging population, scalable interventions, which can be delivered by non-specialized workers are necessary^7,8^.

Metacognitive Training for depression in older adults (MCT-Silver; http://www.uke.de/mct-silver), is a manualized, CBT-based group intervention^9,10^, which is based on Metacognitive Training for Psychosis^11–13^. and Metacognitive Training for Depression (D-MCT)^14^. MCT-Silver adapts a metacognitive perspective (“thinking about thinking”) to encourage “ah ha” experiences, which increase participants’ awareness for unhelpful thoughts, emotion regulation strategies (ER) and information processing biases associated with the onset and maintenance of depression. In a second step, the training encourages participants to identify more helpful, alternative beliefs and thoughts as well as behavioral changes to reduce depressive symptoms. Specifically, MCT-Silver addresses negative beliefs as well as ER strategies (e.g., withdrawal, rumination, acceptance), which have been implicated in depression in numerous studies from basic and translational research^15–20^. Differing from traditional cognitive-behavioral approaches^21^, MCT-Silver also addresses dysfunctional metacognitive beliefs (e.g., “the more I think about my problems, the more likely it is that I will find a solution) as well as information processing biases (e.g., memory preferences for negative information such as when an older adult primarily remembers the days on which their family does not call them). Such information processing biases often occur automatically and may contribute to reinforcing negative beliefs (e.g., “I am not important to my family.”) and depressive symptoms. The eight MCT-Silver sessions (see Online Resource 1) are structured and administered via a multimedia presentation so that delivery requires minimal preparation time and groups can also be conducted by mental healthcare workers without extensive training in psychotherapy (E-learning available at www.uke.de/e-dmct). An open format is used so that participants can join and leave the group at any time. Worksheets summarizing the session content, which also include exercises to work through personal examples are to be completed between sessions. MCT-Silver represents an age-adapted version of Metacognitive Training for Depression (D-MCT)^14^, which has yielded moderate to large effects on depressive symptoms compared to an active control group after eight weeks of treatment as well as at 6-month^22^ and 3.5 year follow-up assessments (secondary assessments)^23^. Differing from D-MCT, MCT-Silver also includes elements of third-wave therapies (e.g., values, acceptance^24^ and imagery rescripting^25^), so that it can be best described as a “modern”, age-adapted CBT-based intervention that utilizes a metacognitive approach. There is initial evidence that MCT-Silver leads to a reduction in depression and rumination among older adults both after 8-weeks of intervention and three months later compared to an active control group^26^.

Despite significant research on CBT interventions^27^, it remains equivocal exactly which processes are associated with change in depression due to CBT-based approaches^28^. Identification of components (e.g., modules for modularized therapies) that lead to symptom change is one approach to improving knowledge about possible processes through which interventions exert their effects [^28^ but see also^8^]. A better understanding of module-specific effects can help identify which intervention content is associated with change in depressive symptoms as well as the specific constructs targeted in the intervention.

Previous work on MCT-Silver and D-MCT provides preliminary evidence for variables through which the trainings may exert their effects (i.e., mediation), as well as session-specific effects for D-MCT. Module-specific effects of MCT-Silver have not yet been examined. For example, Schneider et al.^10^ reported that improvements in rumination and negative cognitive beliefs (measured pre- to post-) mediated improvement on clinician-rated and self-reported depression (pre- to follow-up), respectively, among participants undergoing MCT-Silver (versus cognitive remediation). In similarly designed analyses for D-MCT, metacognitive beliefs pertaining to “need for (cognitive) control” significantly mediated improvement in depressive symptoms^29^, whereas neither negative cognitive beliefs nor positive metacognitive beliefs emerged as significant mediators. Conclusions that can be drawn from these analyses are; however, limited, because analyses concerned overall changes across the course of all modules versus module-specific changes. Analyses of session-specific effects for D-MCT^30^ revealed significant improvements in many variables over the course of treatment (i.e., self-esteem, all-or-nothing thinking, guilt, negative filter, black-and-white thinking, social withdrawal, mind reading, memory deficits, overconfidence in emotion recognition and rumination), as well as within-sessions (i.e., ability to accept praise, overconfidence in emotion recognition, should statements, black-and-white thinking). Some of these changes (i.e., improved energy, confidence in emotion recognition, and all-or-nothing thinking as well as reduced rumination) were also significantly associated with reductions in depressive symptoms.

The current study represents an initial examination of whether change in specific variables within or after modules addressing variable-specific content (e.g., mental filter in Module #1) can be detected and whether change in these variables may also occur over the course of the intervention and are associated with reduction in depressive symptoms. To this end, in a secondary analysis of a randomized controlled trial (RCT)^26^, we evaluated participants’ negative cognitive beliefs (“I only pay attention to the negative details of situation.”), metacognitive beliefs (“Rumination helps me to better organize my thoughts.”), depressive symptoms, and ER strategies (“I tend to withdrawal from other people.”; “I try to accept my negative feelings even when I don’t like them.”), as well as attitudes toward aging (“With age, everything gets worse.”) and self-esteem (“Overall, I am satisfied with myself.”) before and after each session. Our aim is to use this information to modify MCT-Silver modules to improve the efficacy of the intervention. Although we hypothesized that all investigated variables would improve over the period of the intervention, we assumed that within session changes (i.e., pre-post) for each variable would be greatest immediately following the session in which it was targeted (within-session effects; for example, mental filter in Module #1) and one week after that session (between-session effects) compared to after and within all other modules (e.g., for mental filter Modules 2 through 8). In exploratory analyses, we examined whether change (pre-post session) in variables occurring within the specifically targeted session was associated with change in depressive symptoms according to the primary outcome, Hamilton Depression Rating Scale (HDRS)^31^, over the course of the treatment (pre-post). Finally, we sought to identify which modules were assessed most positively by participants and report patients’ appraisal ratings for each module.

## Materials and methods

### Design

The study represents a secondary analysis of an RCT examining the efficacy of MCT-Silver compared to an active control intervention. The study was conducted on an outpatient basis at the University Medical Center Hamburg-Eppendorf. Participants were allocated either to MCT-Silver or the active control intervention on a 1:1 ratio using a computer-generated allocation plan. For a more detailed description of the study, see Schneider et al. From October 2018 to January 2021 participants were assessed at three time points: baseline, post (8 weeks) and follow-up (3 months after post). The trial ended upon recruitment of the planned number of participants. In the present study, we only considered data from the intervention group, and we only analyze data from pre- and post-session questionnaires as well as the change in depressive symptoms from baseline to post-assessment in the MCT-Silver group based on the HDRS. All participants had full access to care as usual (CAU) and provided written informed consent prior to participation in the study. As compensation for the time in completing the assessments, participants received 20 Euros per assessment.

### Participants and procedure

**Figure 1:**
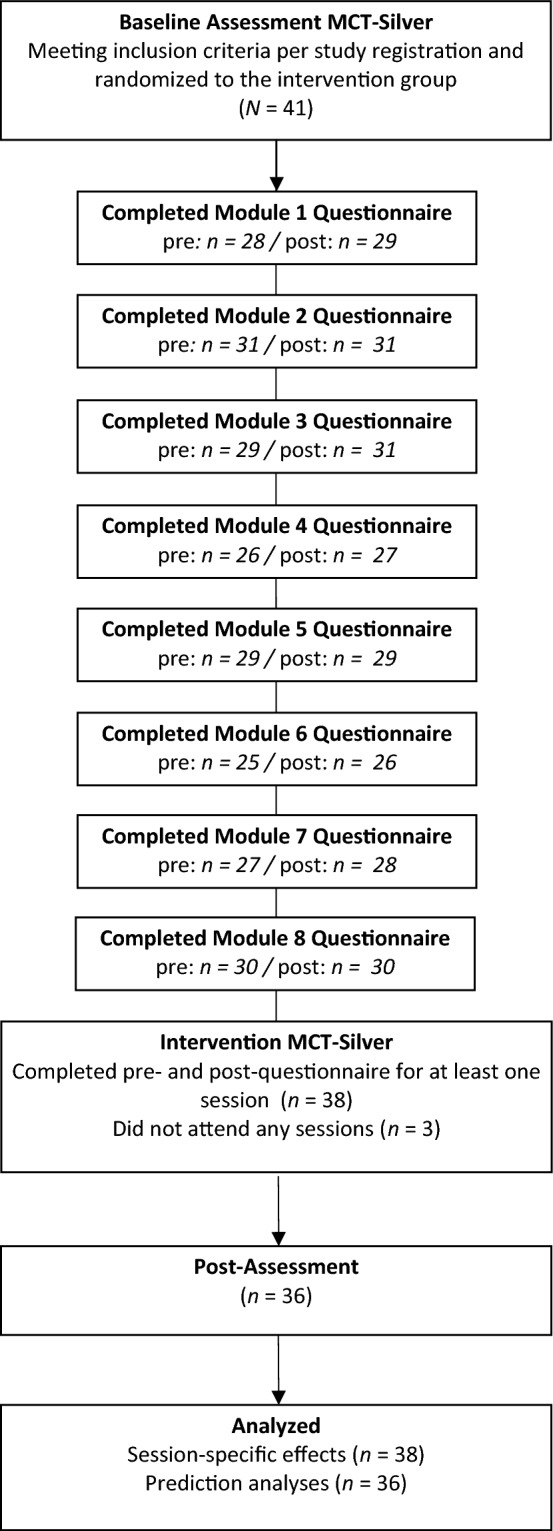
Participant flow chart.

Participants (*N* = 80) were recruited via Google AdWords, articles in a senior magazine, posters, brochures, word-of-mouth, and advertisements, as well as through depression, anxiety and geriatric psychiatry outpatient clinics. The Mini International Neuropsychiatric Interview (MINI; German version, 7.0.2)^32^ was used to assess single-episode or recurrent depression or dysthymia (inclusion criteria), psychiatric comorbidities, and exclusion criteria based on the Diagnostic and Statistical Manual (DSM)-5 criteria. Raters had at least a bachelor’s degree in psychology, were trained prior by the lead investigators (BS, RV) and blinded for randomization/group allocation. Additionally, participants were invited to participate if they were (1) at least 60 years old, (2) provided consent to participate in the study, (3) available for weekly sessions, (4) eligible for group therapy (ability to comply with group rules was assessed during the screening interview), (5) had sufficient German language skills, and (6) scored within the intact range (≥ 17 points) on a telephone version of the Mini Mental State Examination^33^. In line with previous studies^12,22,34^, concurrent outpatient psychotherapy or pharmacological treatment was carefully documented. The exclusion criteria were: (1) lifetime psychotic symptoms, (2) lifetime mania, (3) severe neurological disease (e.g., Parkinson’s disease, multiple sclerosis), (4) current substance dependence, (5) visual or hearing impairment, which prevented group participation and/or testing, and/or (6) current acute suicidality. Current substance use or abuse was tolerated. For the present analyses, only patients who met inclusion criteria of the main study and were randomized to MCT-Silver were considered. See Fig. 1 for the flow chart for the study. Completers and non-completers did not significantly differ based on sociodemographic variables (all *p*’s > 0.13).

### Power analysis

The minimum detectable effect size was estimated using the R package simr^35^. The power analysis indicated that, for the models applied and the current sample, a minimum detectable effect size with a power of 0.80 was small to moderate (Cohen’s *d* = 0.44).

### Measures

The primary outcome for the main RCT study was the Hamilton Depression Rating Scale (HDRS). The HDRS is a semi-structured interview consisting of 17-items assessing depressive symptom severity and frequency over the past week. A total score of eight or less indicates the absence of clinically relevant depression^36^. The HDRS demonstrates good internal consistency (Cronbach’s alpha = 0.78)^37^ and test–retest reliability (*r* = 0.93).

### Pre- and post-session questionnaire

Before and after each MCT-Silver session, participants were asked to complete a short questionnaire assessing depressive symptoms, negative cognitive beliefs, self-esteem, metacognitive beliefs, and ER strategies (see Online Resource 2). The questionnaires included 18 items, which had to be answered on a 5-point Likert scale (1 = *fully disagree* to 5 = *fully agree*). Four items were inverse and needed to be recoded. Specifically, many of the questions (i.e., items 5, 6, 7, 9, 10, 11, 12, 14, 15, 16, 17, 19 and 20) were drawn directly from a questionnaire developed for an RCT on D-MCT^30^. Two questions were rephrased for the current study (i.e., items 3 and 13). Reflecting MCT-Silver content that differs from D-MCT, new questions regarding attitudes toward aging and acceptance and values were developed (i.e., items 4, 8 and 18) and were drawn directly from content presented in the multimedia presentation. For example, in module 8, there is an exercise in which participants are asked to generate ideas regarding the positive aspects of aging. Thus, we developed the item “With age, everything becomes worse,” to correspond directly to this exercise. To reduce participant burden, most domains were assessed by one item each (i.e., worry about memory, acceptance, black-and-white thinking, values, rumination, social withdrawal, fortune-telling, mind-reading, aging, mental filter, overgeneralization), but two were assessed by two items (self-esteem and attributions). Depressive symptoms were assessed by three items. Thus, 14 session variables were used for the analyses.

### Subjective appraisal ratings

After each MCT-Silver session, participants answered two items regarding the subjective appraisal of the session: (1) “The training session was useful” and (2) “I want to apply the lessons learned in my everyday life”. The items had to be answered on a 5-point Likert scale (1 = *completely disagree* to 5 = *completely agree*). The two items were based on the Subjective Appraisal Rating Scales that were already used in other studies on MCTs^22,34^.

### Data analyses

Data analyses were based on that of Miegel et al.^30,38^. The session effects were analyzed in three ways: calculating the (1) change of variables over the course of the sessions, (2) the change of variables within one session (i.e., from before to after one session), and (3) the change of variables between two sessions (i.e., from after one session to the start of the next session). For the first set of analyses (i.e., the change over the course of the MCT-Silver) the following three analyses were computed as change in the variables might be different over the course of the treatment: (1) *the rate of change* in each variable *per module*, from the first to the last session (i.e., coding the sessions from 0 [first session] to 7 [last session]); (2) the immediate effect for each construct from before to after its corresponding module (i.e., coding the sessions before the module of interest with a 0 and after the module of interest with a 1; *elevation post module*), and (3) the gradual effect of a module which can be seen in the change of the time slope from before to after the module (i.e., coding the sessions before the module of interest with a 0, the post-assessment for module with a 0, the subsequent module with a 1, the subsequent module with 2 and so on, until the last module; *rate of change post module*).

Data was prepared using SPSS version 27, and linear mixed models as well as prediction analyses using lasso regression were computed in R studio version 1.2.5033^39^. In a sensitivity analysis, a mediation model was tested using the SPSS PROCESS macro^40^. LMMs were used for analyzing session effects as data were hierarchically structured with repeated measures by the pre- and post-session questionnaires on level 1 and patients on level 2. Either a random intercept or random slope, random intercept model was used for analyses depending on the best model fit, which was identified by comparing both models by ANOVA’s. As we wanted to choose a parsimonious model, a random intercept, random slope model was only used if it significantly improved model fit, thus, if the models significantly (*p* < 0.05) differed based on the ANOVA. Effect sizes can be interpreted similarly to that of Cohen’s* d* with 0.2 indicating a small, 0.5 indicating a medium, and 0.8 indicating a large effect and, in line with previous work^30,38,41^ were computed by dividing the beta value of any variable of the pre- and post-session questionnaire by the standard deviation of that pre variable. Although we hypothesized that significant changes would be detected for specific variables within (pre-post) and the week following their corresponding modules (post–pre; e.g., mental filter would change within module #1 addressing mental filter as well as and during the week after module #1), we also examined on an exploratory basis if change in each of the 13 variables could be detected for each module. Exploratory hypotheses (i.e., 7 for each variable) needed to withstand a Bonferroni correction (i.e.,* p* < 0.007).

For the identification of predictors for the change in depressive symptoms (pre-post HDRS) lasso regression analyses were performed. For this, difference scores in the pre-post-session questionnaire variables served as independent variables and the difference score in the HDRS served as the dependent variable. All difference scores were divided by the baseline score of the respective variable and multiplied by 100 to receive a percentage change in variables. For the independent variables, only those variables that were hypothesized to change in a particular module (e.g., mental filter in module #1) were tested as possible predictors, except for the change in depression for which changes in all modules were used for exploratory purposes, resulting in 21 possible predictors. Missing values in the independent variables were imputed by using the random forest R package^42^. We used a tenfold cross-validated lasso regression^43^ by setting the alpha to 1. Lambda was determined via cross-validation in glmnet function by repeated *k*-fold cross-validation, which has the advantage of improving the estimated performance of the machine learning model. The predictors that were identified by lasso regression were entered into a linear regression analysis for validation objectives. Finally, a sensitivity analysis testing whether change in mental filter (pre-post Module 1; M) mediated baseline symptom score (X) predicting post-treatment symptom score (Y) was conducted. To correct for potential biases of non-normality in the sample, results were bootstrapped 5,000 times. When the effect range (LL = lower limit to UL = upper limit) of the 95% CI does not include zero, the null hypothesis is considered rejected.

### Statement of ethics

This study protocol was reviewed and approved by the Local Psychology Ethics Commission (Lokale Psychologische Ethikkomission) of the Center for Psychosocial Medicine at the University Medical Center Hamburg-Eppendorf, approval number (LPEK-005). All participants provided written informed consent prior to participation in the study. The study was registered at ClinicalTrials.gov (NCT03691402) and conducted in accordance with the Declaration of Helsinki.

## Results

### Sociodemographic and psychopathological variables

See Table 1 for baseline demographic data and changes in medication as well as depressive symptoms from baseline to post-assessment of the patients who participated in post-assessment and completed a questionnaire pre- and post- for at least one MCT-Silver module. Thirty-eight participants were included in the analyses of the session effects and *n* = 36 in the analyses for the lasso regression; *n* = 2 dropped out for post-assessment. The overall rate of missingness was 18%. In line with previous reports^10,26^, participants had moderate depressive symptom severity (HDRS) at baseline, which declined with large effect sizes from baseline to post-assessment (*t*[35] = 5.93, *p* < 0.001, *d* = 1.31). There were also significant changes in medications such that fewer participants were taking anti-depressants (*n* = 4) or benzodiazepines (*n* = 1) at post-intervention (all *p*’s < 0.001). The following comorbid diagnoses were identified using the M.I.N.I.: obsessive–compulsive disorder (*n* = 1); alcohol-related disorder (last 12 months; *n* = 1); panic disorder (lifetime; *n* = 3); agoraphobia (current, *n* = 6); social phobia (current, *n* = 2); generalized anxiety disorder (current, *n* = 5).

**Table 1:** Demographic, symptom, and treatment data: Mean (M), standard deviation (*SD*), frequency (n) and percent (%).

	Baseline (*N* = 38)	Post (*n* = 36)	Statistics
	*M* or *n/SD* or %	*M/SD*	
Age (years)	72.18 (6.36)	–	
Sex (m/f)	10 (26.3%) 28 (73.7%)	–	
Years of formal education	10.11 (1.78)	–	
Depressive episode (M.I.N.I)		–	
Current	12		
Past (lifetime)	11		
Past and current episode	14		
None	1		
Number of depressive episodes	3.36 (4.32)	–	
Dysthymia (current)	17 (44.7%)	–	
Number of MCT-Silver sessions	6.16 (2.09)	–	
Medication			
Antidepressant	14^a^	10	$\chi$^2^ (1) = 21.76, *p* < 0.001
Benzodiazepine	4	3	$\chi$^2^(1) = 26.18, *p* < 0.001
None	23	25	$\chi$^2^(1) = 22.18, *p* < 0.001
HDRS	17.23 (5.03)	10.31 (5.58)	*t*(35) = 5.93, *p* < 0.001,* d* = 1.31

*HDRS* Hamilton Depression Rating Scale. ^a^*n* = 3 participants at baseline and *n* = 3 participants post-intervention were taking both benzodiazepines and anti-depressants.

### Ratings at baseline

At baseline, participants endorsed moderate ratings on most items indicating that they did not fully agree or disagree with the statements (values: *M* = 3.17, *SD* = 1.32; attribution: *M* = 3.13, *SD* = 0.84; memory: *M* = 3.03, *SD* = 1.42; self-esteem: *M* = 3.00, *SD* = 1.05; rumination: *M* = 2.83, *SD* = 1.13; acceptance: *M* = 2.78, *SD* = 1.23; mind reading: *M* = 2.70, *SD* = 1.29; black-and-white thinking: *M* = 2.61, *SD* = 1.18; fortune telling: *M* = 2.59, *SD* = 1.29 and overgeneralization: *M* = 2.49, *SD* = 1.35). However, participants more strongly endorsed items regarding mental filter (*M* = 3.89, *SD* = 1.02), depressive symptoms (*M* = 3.75, *SD* = 0.98), social withdrawal (*M* = 3.68, *SD* = 1.31), and aging (*M* = 3.49, *SD* = 1.12).

### Changes over the course of the treatment

Due to a better model fit, the random intercept model was chosen for most analyses. Depression (*b* = − 0.09 [95% CI − 0.14, − 0.04], *p* = 0.001, Cohen’s *d* = 0.08) and mental filter (*b* = − 0.12 [95% CI − 0.20, − 0.04], *p* = 0.004, Cohen’s *d* = 0.12) improved significantly over the course of the treatment with small effect sizes. Black-and-white thinking improved from the time the module (module #3) was delivered to the last MCT-Silver session with a small effect size (*b* = − 0.33 [95% CI − 0.66 − 0.01], *p* = 0.045; Cohen’s *d* = 0.27). All other variables did not change significantly (see Table 2).

**Table 2:** Results of the rate of change per module, the elevation after the module and the rate of change after the module.

Variable	b	95% CI	*p*
Depression			
Rate of change per module	− **0.09**	− **0.14 to **− **0.04**	**0.001**
Mental filter			
Rate of change per module	− **0.12**	− **0.20 to **− **0.04**	**0.004**
Elevation post module	0.07	− 0.26 to 0.41	0.661
Rate of change post module	0.10	− 0.01 to 0.21	0.081
Overgeneralization			
Rate of change per module	0.04	− 0.09 to 0.16	0.704
Elevation post module	0.07	− 0.23 to 0.38	0.637
Rate of change post module	0.04	− 0.09 to 0.16	0.557
Memory			
Rate of change per module	0.01	− 0.09 to 0.12	0.804
Elevation post module	− 0.17	− 0.63 to 0.30	0.487
Rate of change post module	0.06	− 0.10 to 0.22	0.454
Acceptance			
Rate of change per module	0.04	− 0.03 to 0.12	0.251
Elevation post module	− 0.37	− 0.76 to 0.02	0.060
Rate of change post module	0.05	− 0.09 to 0.20	0.472
Black and white thinking			
Rate of change per module	0.03	− 0.05 to 0.11	0.472
Elevation post module	− **0.33**	− **0.66** to − **0.01**	**0.045**
Rate of change post module	0.08	− 0.07 to − 0.22	0.310
Values			
Rate of change per module	− 0.06	− 0.15 to 0.02	0.131
Elevation post module	0.11	− 0.31 to 0.52	0.607
Rate of change post module	− 0.06	− 0.20 to 0.07	0.360
Attribution			
Rate of change per module	− 0.05	− 0.11 to 0.01	0.102
Elevation post module	0.17	− 0.08 to 0.42	0.192
Rate of change post module	0.00	− 0.10 to 0.10	0.940
Rumination			
Rate of change per module	− 0.03	− 0.10 to 0.04	0.387
Elevation post module	− 0.07	− 0.40 to − 0.27	0.696
Rate of change post module	0.05	− 0.06 to 0.16	0.383
Social withdrawal			
Rate of change per module	− 0.04	− 0.13 to 0.05	0.337
Elevation post module	0.02	− 0.28 to 0.33	0.889
Rate of change post module	− 0.00	− 0.14 to 0.13	0.958
Fortune telling			
Rate of change per module	0.06	− 0.03 to 0.14	0.208
Elevation post module	− 0.22	− 0.54 to 0.10	0.183
Rate of change post module	− 0.04	− 0.18 to 0.09	0.525
Mind reading			
Rate of change per module	− 0.01	− 0.07 to 0.05	0.777
Elevation post module	− 0.04	− 0.32 to 0.23	0.770
Rate of change post module	0.01	− 0.09 to 0.10	0.856
Aging			
Rate of change per module	0.01	− 0.04 to 0.06	0.797
Elevation post module	− 0.10	− 0.34 to 0.14	0.417
Rate of change post module	− 0.02	− 0.10 to 0.07	0.719
Self-esteem			
Rate of change per module	− 0.05	− 0.10 to 0.00	0.068
Elevation post module	− 0.14	− 0.40 to 0.12	0.301
Rate of change post module	0.03	− 0.06 to 0.13	0.469

Random intercept model: mental filter, memory, acceptance, values, rumination, mind reading, aging, self-esteem; Random intercept, random slope model: depression, overgeneralization, black-and-white thinking, attribution, social withdrawal, fortune telling.

Significant changes are set in bold.

### Within-session changes

For most analyses, the random intercept model provided a better model fit than the random intercept, random slope model (see Table 3 for details). Two variables changed more in one module compared to all other modules with small to medium effect sizes: Overgeneralization worsened more after module #1 (Mental Filter; Cohen’s *d* = 0.27) and rumination improved more after module #6 (Rumination/Social Withdrawal; Cohen’s *d* = 0.44). Some other variables (e.g., black-and-white thinking) also changed more in one module (e.g., module #1) compared to others, but these exploratory analyses did not withstand the Bonferroni corrections.

**Table 3:** Results of the linear mixed-effects model for all modules, within-session changes, presented as beta value for fixed effect (*B*).

Module	DV1	DV2	DV3	DV4	DV5	DV6	DV7	DV8	DV9	DV10	DV11	DV12	DV13	DV14
Range (Intercept)	0.49–0.52	2.69–2.74	0.92–1.03	1.95–2.05	1.71–1.81	0.71–0.84	1.31–1.37	0.64–0.79	1.27–1.40	0.77–1.04	1.07–1.12	1.33–1.40	0.35–0.87	1.39–1.40
Module 1	0.11	− 0.07	0.34*	− 0.09	− 0.10	0.46	0.22	0.13	0.31	0.16	− 0.06	0.20	0.05	0.01
Module 2	0.06	− 0.05	− 0.00	0.23	0.11	− 0.18	0.04	0.15	− 0.14	0.07	− 0.04	0.12	− 0.27	− 0.05
Module 3	− 0.11	0.12	− 0.00	− 0.04	− 0.22	0.22	− 0.07	0.05	0.06	− 0.03	0.12	− 0.08	0.04	0.01
Module 4	0.01	0.20	0.01	0.39	− 0.16	− 0.07	− 0.11	− 0.05	0.25	0.04	− 0.08	− 0.01	0.02	− 0.01
Module 5	0.06	0.01	0.00	0.02	0.06	0.02	− 0.16	− 0.12	0.25	− 0.05	− 0.31	− 0.19	− 0.11	0.08
Module 6	− 0.05	− 0.30	− 0.20	0.03	0.15	− 0.19	0.01	− 0.13	− 0.48***	− 0.12	0.21	0.03	0.11	− 0.03
Module 7	0.00	0.21	− 0.07	− 0.16	0.18	− 0.25	0.11	− 0.02	0.00	0.02	0.12	− 0.11	0.10	− 0.06
Module 8	− 0.08	− 0.12	− 0.07	0.12	− 0.01	− 0.05	− 0.02	0.00	− 0.26	− 0.13	0.07	0.03	0.01	0.04
Random Parts														
Time	0.25	0.57–0.58	0.27–0.32	0.84–0.85	0.54–0.55	0.49–0.53	0.42–0.49	0.24–0.28	0.36–0.46	0.32–0.33	0.37–0.45	0.32	0.29–0.34	0.18
Patients	0.10	0.11	0.16–0.19	0.38–0.41	0.15–0.19	0.03–0.08	0.36–0.45	0.04–0.08	0.22–0.28	0.12–0.20	0.21–0.24	0.46–0.49	0.08–0.11	0.34
ICC	0.28	0.16–0.17	0.36–0.44	0.31–0.33	0.25–0.26	0.13–0.17	0.42–0.52	0.13–0.29	0.32–0.47	0.35–0.39	0.31–0.42	0.59–0.61	0.20–0.33	0.66
Nid	38	37	38	38	38	38	38	38	38	38	37	38	37	38
Observations (*N*)	225	216	220	219	212	216	213	224	216	217	215	218	217	222

DV1 = Depression; DV2 = Mental Filter; DV3 = Overgeneralization; DV4 = Memory; DV5 = Acceptance; DV6 = Black and White Thinking; DV7 = Values; DV8 = Attribution; DV9 = Rumination; DV10 = Social Withdrawal; DV11 = Fortune Telling; DV12 = Mind Reading; DV13 = Aging; DV14 = Self-esteem; Module 1 = Mental Filter; Module 2 = Mood-congruent memory/false memories; Module 3 = “should” statements/disqualifying the positive/acceptance of negative feelings; Module 4 = Values; Module 5 = Exaggeration/Minimization/Attribution style; Module 6 = Rumination/social withdrawal; Module 7 = Jumping to conclusions; Module 8 = Self-worth in later life. For DV3 (module 1), DV5 (module 5), DV6 (module 7), DV7 (module 4), DV8 (module 1 & 6), DV9 (module 7), DV10 (module 6), DV11 (module 5), and DV13 (module 3 & 5) the random intercept, random slope model was used; all other LMMs were conducted with a random intercept model.

*ICC* Intraclass correlations; *p < 0.05, **p < 0.01, ***p < 0.007 (Bonferroni correction). Items were rated on a Likert scale (1 = *fully disagree* to 5 = *fully agree*) and, when necessary, items were reverse scored prior to analysis.

### Between-session changes

For the analyses of all variables of module #8 the random intercept, random slope model was used, and for all other analyses, the random intercept model provided a better model fit. Except for one exploratory result that did not withstand Bonferroni correction (improvement in depression one week after module #1; see Table 4 for details) no significant between-session change was found.

**Table 4:** Results of the linear mixed-effects model for all modules, between-session changes, presented as beta value for fixed effect (*B*).

Module	DV1	DV2	DV3	DV4	DV5	DV6	DV7	DV8	DV9	DV10	DV11	DV12	DV13	DV14
Range (intercept)	1.83–2.16	2.62–2.71	0.74–1.02	1.97–2.10	1.35–1.45	1.76–2.08	1.27–1.68	1.22–1.74	1.47–1.71	1.04–1.53	1.03–1.56	0.92–1.30	1.23–2.91	1.07–1.12
Module 1	− 0.38	− 0.02	− 0.26	0.01	− 0.12	− 0.06	− 0.11	− 0.28	− 0.33	− 0.26	− 0.19	− 0.34	0.13	− 0.07
Module 2	0.14	0.21	0.32	− 0.03	0.12	0.25	− 0.01	0.11	0.14	− 0.02	0.19	− 0.02	0.11	− 0.14
Module 3	0.11	0.13	− 0.17	0.08	− 0.36	− 0.33	0.44	− 0.28	0.33	0.05	− 0.04	0.07	− 0.02	− 0.08
Module 4	− 0.09	− 0.02	− 0.03	0.08	− 0.03	− 0.19	0.41	0.19	− 0.18	− 0.01	0.14	− 0.03	− 0.10	0.03
Module 5	− 0.05	− 0.07	− 0.02	− 0.07	0.11	0.03	− 0.31	0.13	− 0.10	0.17	0.09	0.18	0.05	− 0.12
Module 6	− 0.23	0.10	0.07	− 0.25	− 0.19	− 0.06	− 0.21	0.02	− 0.03	− 0.07	− 0.00	− 0.08	− 0.14	− 0.03
Module 7	0.02	− 0.26	0.18	0.13	0.23	0.22	− 0.05	− 0.12	0.12	− 0.23	− 0.29	0.08	− 0.05	0.18
Module 8	0.40	0.19	− 0.10	0.06	0.15	0.07	− 0.15	0.29	0.02	0.19	0.19	0.25	− 0.01	0.13
Random parts														
Time	0.30–0.54	0.53–0.67	0.51–0.88	1.08–1.35	0.70–0.82	0.40–0.81	0.83–1.11	0.31–0.49	0.53–0.66	0.43–0.91	0.61–1.06	0.38–0.92	0.26–0.89	0.40–0.79
Patients	0.15–0.38	0.15–0.22	0.01–0.22	0.14–0.31	0.03–0.12	0.35–0.74	0.06–0.27	0.00–0.14	0.16–0.29	0.00–0.32	0.02–0.43	0.01–0.28	0.00–0.81	0.00–0.09
ICC	0.22–0.60	0.18–0.36	0.02–0.46	0.10–0.28	0.04–0.19	0.30–0.68	0.05–0.31	0.40	0.20–0.38	0.53	0.01–0.49	0.01–0.61	0.77	0.49
Nid	35	35	34	35	35	35	35	35	35	35	35	35	35	35
Observations (*N*)	185	179	180	180	174	177	173	185	178	177	179	178	179	184

DV1 = Depression; DV2 = Mental Filter; DV3 = Overgeneralization; DV4 = Memory; DV5 = Acceptance; DV6 = Black and White Thinking; DV7 = Values; DV8 = Attribution; DV9 = Rumination; DV10 = Social Withdrawal; DV11 = Fortune Telling; DV12 = Mind Reading; DV13 = Aging; DV14 = Self-esteem; Module 1 = Mental Filter; Module 2 = Mood-congruent memory/false memories; Module 3 = “should” statements/disqualifying the positive/acceptance of negative feelings; Module 4 = Values; Module 5 = Exaggeration/Minimization/Attribution style; Module 6 = Rumination/social withdrawal; Module 7 = Jumping to conclusions; Module 8 = Self-worth in later life. For DVs 1–14 (module 8) the random intercept, random slope model was used; all other LMMs were conducted with a random intercept model. Items were rated on a Likert scale (1 = *fully disagree* to 5 = *fully agree*) and, when necessary, items were reverse scored prior to analysis.

ICC = Intraclass correlations.

### Prediction analyses

The lasso regression revealed four predictive variables for the change in depressive symptoms (HDRS) from baseline to post-assessment: a worsening in depression in module #2 (− 0.03) and module #4 (− 0.08), a worsening in memory in module #2 (− 0.27), and an improvement in mental filter in module #1 (0.20). To validate the identified predictors, they were entered into a linear regression analysis. Only the improvement in mental filter in module #1 was found to be a significant predictor for the change in the HDRS (β = 0.57, *p* = 0.002). This predictor explained 30.2% of the change in the HDRS.

### Sensitivity analysis

The indirect effect of baseline depressive symptoms on post-intervention symptoms through change in mental filter in Module #1 (b = − 0.07, SE = 0.07, BootLLCI = − 0.22, BootULCI = 0.033) was not significant. Only the association between post-intervention HDRS score and change in mental filter (path b) after entry of HDRS baseline score reached significance (*b* = − 0.03, *SE* = 0.01, *p* = 0.02; BootLLCI = − 0.06, BootULCI = − 0.01; *R*^2^ = 0.26, *F*(2, 24) = 4.17).

### Subjective appraisal of the modules

Overall, participants’ appraisal of the modules was mostly positive with the least positive rating regarding the usefulness of module #1 (71.5% agree/completely agree). Module #3 was evaluated most positively (see Table 5).

**Table 5:** Subjective appraisal (%) of MCT-Silver modules.

Module	Completely disagree	Mostly disagree	Neither agree nor disagree	Mostly agree	Completely agree
“I want to apply the lessons I learned in my everyday life.”					
1	0.0	2.6	8.3	54.2	33.3
2	0.0	0.0	7.4	51.9	40.7
3	0.0	0.0	0.0	54.8	45.2
4	0.0	4.2	12.5	50.0	33.3
5	0.0	0.0	14.3	35.7	50.0
6	0.0	4.3	4.3	56.5	34.8
7	0.0	0.0	3.8	57.7	38.5
8	0.0	3.6	7.1	35.7	53.6
“The session was useful.”					
1	0.0	7.1	21.4	42.9	28.6
2	0.0	0.0	12.9	45.2	41.9
3	0.0	6.5	3.2	35.5	54.8
4	0.0	8.3	8.3	41.7	41.7
5	0.0	0.0	10.3	51.7	37.9
6	0.0	2.6	7.7	53.8	34.6
7	3.6	0.0	14.3	39.3	42.9
8	0.0	3.3	10.0	30.0	56.7

## Discussion

The present study represents an initial trial aiming to identify changes in module-specific variables across the eight modules of MCT-Silver, in- and between-session effects, and correlates of change in depressive symptoms in a small sample of older adults. We also report subjective appraisal of specific sessions. It should be emphasized that the questionnaire utilized to identify session-specific effects measured the variables of interest primarily with one to two items, which is a methodological limitation and should be considered in the interpretation of our findings as this may have limited detection of changes^38^.

In line with our hypotheses, significant (though small) changes across treatment for depressive symptoms and mental filter as well as persistent reductions in black-and-white thinking following module #3 were detected. Broadly in-line with these module-specific effects, we previously reported^26^ small (Cohen’s *d* = 0.30) but significant reductions in negative cognitive beliefs^44^. However, differing from this work, based on a single item used in the current questionnaire, there were no significant changes across all sessions in the positive metacognitive belief assessed (i.e., “Rumination helps me to better organize my thoughts.”). Additionally, we found no significant changes for other negative cognitive beliefs (i.e., overgeneralization, memory, attribution, fortune-telling, mind-reading), ER strategies (i.e., acceptance, withdrawal), attitudes toward aging or self-esteem across treatment.

Effects on depression (as assessed by three items) were small, and thus diverge from the primary findings of this RCT indicating large reductions on the HDRS^26^. Nonetheless, the current findings provide initial evidence that MCT-Silver meets its main aims of reducing depressive symptoms and (specific) negative cognitive beliefs. Particularly, content regarding mental filter and black-and-white thinking is relevant and was conveyed effectively. Nonetheless, which specific MCT-Silver elements may be associated with a reduction in negative mental filter and black-and-white thinking remain somewhat unclear. In mediation studies, it would be interesting to examine whether the information presented in the introductory slides for each session, which introduce the meta-perspective utilizing a metaphor, leads to a reduction in mental filter across sessions. Additionally, in several exercises throughout the training, participants practice finding alternative, more positive, thoughts and explanations for (negative) situations or thoughts. This may have generalized to an improvement of focus on neutral or positive aspects of situations. It would also be of interest to examine more specifically which aspects of module #3 may contribute to a lasting reduction in black-and-white thinking, especially given that the in- and between-session analyses did not indicate that any one module was associated with a significantly greater reduction in this variable. Studies of single sessions focusing on specific MCT-Silver content or techniques (e.g., practice with findings alternative thoughts vs. gaining distance from thoughts) could further help differentiate these session- and even technique and content-specific effects^8^.

Results of the within-session analyses also only partially confirmed our hypotheses such that changes in the specific element targeted were found for two modules. Whereas overgeneralization worsened more during module #1 (Mental Filter), rumination improved significantly during module #6 (Rumination/Social Withdrawal) at a small to moderate effect. Significant change for other variables either within their specific modules or the other modules was not detected. Since overgeneralization was only measured with one item, future studies utilizing several items are needed to confirm the increase within module #1. Although the worsening of overgeneralization (“When a mishap occurs, this shows that actually everything always goes wrong for me.”) may suggest unwanted treatment effects, it may also reflect patients’ increased awareness for the extent to which they hold negative cognitive beliefs. Indeed, increased insight is one of the goals of CBT approaches, including MCT^21^. In session of negative cognitive beliefs has been observed in other trials of MCT^41^ as well as for depressive symptoms in other interventions^45^. Moreover, sustained increases in overgeneralization were not detected over the course of the intervention. Momentary worsening may also indicate frustration with a module, lack of mastery of the concepts introduced or lack of alignment on components of treatment. The reduction of rumination within module #6 is generally commensurate with our previous work^26^, indicating reduced positive metacognitive beliefs regarding the usefulness of rumination in the MCT-Silver group. Although, we also found that rumination as measured by the short-form version of the Ruminative Response Scale^46^ significantly decreased over the course of treatment and mediated the reduction in depressive symptoms^10^, this scale includes items regarding ruminative thought content (e.g., I think “Why can’t I handle things better?”) or concrete behaviors rather than metacognitive beliefs about rumination (as assessed in the present study). Previous D-MCT studies have included an item corresponding to engagement in rumination (“I often engage in rumination.”)^30^. Thus, in future work on MCT-Silver, it is important to add items to our questionnaire that measure rumination directly.

Regarding variables associated with a reduction in depressive symptoms from pre- to post-intervention, although several variables were initially identified (e.g., worsening in depression, worsening in memory), only the improvement in mental filter in module #1 remained significantly associated with reduced depressive symptoms and accounted for approximately 30% of the change in depressive symptoms. In a sensitivity analysis, mental filter did not significantly mediate baseline depressive symptoms predicting post-intervention depressive symptoms. Nonetheless, this study provides first evidence that mental filter may be an important variable associated with depression reduction in MCT-Silver. Extending upon this work, it should be considered whether and how mental filter may be more extensively measured to potentially better capture changes in this construct and to detect possible mediation effects.

In line with some previous MCT studies examining between-session effects in outpatient samples^30^. including one study utilizing a revised version of the in-session questionnaire including several items to measure each construct^37^, we did not detect any between-session effects. The two studies that did find between-session effects for MCT for obsessive–compulsive disorder and psychosis were conducted with inpatients and, therefore, it is not possible to differentiate whether other treatments offered in the context of the inpatient stay may have been responsible for between-session changes^41,47^. Somewhat promisingly, in the present study there was also no evidence for deterioration between sessions (e.g., due to frustration with intervention content or homework). Between-session change can also be encouraged by increasing trainers’ emphasis on adherence to homework and discussing homework at the beginning of each session or by encouraging participants to actively apply the skills learned (either formally or informally) between sessions.

Given that we did not find significant reductions in negative beliefs about aging and social withdrawal, intervention content on these areas may need to be strengthened in the future should significant effects not be detected upon improvement of the questionnaire. Secondly, there was no reduction in social withdrawal (“I tend to withdraw from others.”); however, improvements in social withdrawal were found in previous D-MCT studies^30^. The lack of change in social withdrawal is somewhat surprising given that many participants subjectively reported enjoying the social contact the group provided and some indicated that group participation was their only form of social contact. This non-significant effect may reflect the suboptimal phrasing of the item given that avoidance (i.e., a behavior) cannot change within a session. Thus, the questionnaire items for social withdrawal could be revised to include rather beliefs regarding social withdrawal (e.g., reverse scored item, “It is helpful to avoid contact with others when I have a bad mood.”), which are more likely to change over the course of a module and represent a primary target of MCT-Silver.

Participant ratings of specific modules shed light on perceived usefulness and applicability of the module content to daily life. Although module #1 (mental filter) was rated as the least useful module, approximately 87% of participants indicated that they would like to apply the lessons learned in their daily life. Given that reduction in mental filter was the only variable significantly associated with the reduction in depressive symptoms, this incongruence is interesting; it may be more useful to obtain ratings for specific content in modules (i.e., slides on specific variables) versus entire modules.

Although findings cannot be directly compared to those from D-MCT given differences in intervention content and participant characteristics, it is worth noting that improvements in negative filter, black-and-white thinking, overgeneralization and depressive symptoms were also detected in D-MCT studies utilizing similar sample sizes^30^. However, in this work, improvements in variables regarding attributions (module #5), social withdrawal and (module #6), and mind reading/catastrophizing (termed fortune-telling in 30; module #7) were also detected. The content of these D-MCT modules is very similar to the corresponding MCT-Silver modules, and the questionnaire items used in the present study and the previous D-MCT studies are identical. Whereas reductions in memory (module #2) and rumination (module #6) have also been reported for D-MCT, the items for memory and rumination differed from items used in the present study and are, thus, not comparable. Moreover, it should be noted that in the present study, overgeneralization (labeled all-or-nothing thinking in^30^)  was assigned to module #1, whereas in D-MCT studies, this belief was included in analyses for module #3^30^. There are also differences regarding elements (e.g., overgeneralization, lack of energy) associated with reduction in depression symptoms (pre-post intervention). However, in the D-MCT studies, depression was measured by the PHQ-9 and the Beck Depression Inventory-II.

Further work is needed to establish the replicability of the present findings given the small sample size, missing data and use of single items to measure entire constructs. Evidence from Miegel et al.^38^ suggests that revision of the questionnaire so that each module-specific construct is represented by at least three items may lead to improved ability to detect change. Partly to reduce burden on participants, we did not include items corresponding to all MCT-Silver content (e.g., should statements, ruminative behaviors); however, as a result some possible intervention elements remain unexamined. Similarly, particularly the item for memory (“I have no or few worries about my memory”) does not correspond well to the MCT-Silver content and may have been confusing for participants. Refinement of the training content to better target the topics addressed in the MCT-Silver modules (e.g., aging beliefs) may also be necessary if non-significant effects remain after questionnaire improvement. Additionally, according to our power analysis, we were only able to detect effects at *d* $\ge$ 0.44. In our previous work, session-specific changes were often smaller^30,38^. Also, although there are no clear cutoffs regarding amount of missing data^48^ and we used recommended approaches to handle missing data^49^, efforts should be taken to improve data retention in future studies. Additionally, a nested cross-validation machine learning approach could be considered for future work as it can reduce information leakage^50^. Finally, because our study covers only the eight-week timeframe of the intervention, it remains unclear whether sustained effects may have continued after the end of treatment.

Taken together, this study provides many insights regarding module-specific changes in (meta)cognitive and negative aging-related beliefs, behaviors and ER strategies associated with depression, which are purported targets of MCT-Silver. Specifically, we report initial evidence of reductions in mental filter and black-and-white thinking across sessions as well as a module-specific reductions in a positive metacognitive belief regarding rumination among participants completing MCT-Silver. Additionally, there was a session-specific increase in overgeneralization. We plan to use this information to revise the in-session questionnaire. Further research with adequate sample sizes is needed to more definitively examine session-specific and mediation effects.

### Supplementary Information

Supplementary Information 1.

Supplementary Information 2.

Supplementary Information 3.

## Data Availability

The data that support the findings of this article are available from the corresponding author (BS) upon reasonable request.
