# Supplementary material for: Change in negative mental filter is associated with depression reduction in metacognitive training for depression in older adults (MCT-Silver)
Source: Sci Rep. 2024 Jul 25;14:17120. doi: 10.1038/s41598-024-67063-0 (PMC11272923; doi:10.1038/s41598-024-67063-0)
Supplement: Supplementary file 2 — Supplementary Information 2. [file 41598_2024_67063_MOESM2_ESM.docx]

| Item | Topic | Module |
| --- | --- | --- |
| 1. I found the module to be useful. (post only) | Session rating | --- |
| 1. The module helped me to better cope with my disorder. (post only) | Session rating | --- |
| 1. I do not worry (at all) about my memory. | Memory | 2 |
| 1. I try to be practice acceptance of negative feelings, even when I don’t like them. | Acceptance | 3 |
| 1. I only pay attention to the negative details of a situation. | Mental Filter | 1 |
| 1. When a mishap occurs, this shows that actually everything always goes wrong for me.* | Overgeneralization | 1 |
| 1. Lack of energy and motivation, feeling tired | Depressive symptoms | --- |
| 1. My values and goals in life are clear to me. | Values | 4 |
| 1. I feel that I am a loser or worthless. | Self-esteem | 8 |
| 1. Overall, I am satisfied with myself. | Self-esteem | 8 |
| 1. If I don’t complete a task perfectly, this means that I failed. | Black-and-white thinking | 3 |
| 1. If something doesn’t go well, I assume that I am the problem. | Attributional style | 5 |
| 1. Ruminating helps me to better organize my thoughts. | Rumination | 6 |
| 1. Down, sad or hopeless. | Depressive symptoms | --- |
| 1. I tend to avoid other people. | Withdrawal | 6 |
| 1. I expect the worst. | Catastrophizing | 7 |
| 1. I have the feeling that other people think negatively of me. | Mind reading | 7 |
| 1. With age, everything becomes worse. | Aging | 8 |
| 1. If something goes well, this is usually due to chance. | Attributional style | 5 |
| 1. Lack of interest, lack of pleasure | Depressive symptoms | --- |

S2. Pre- and post-session questionnaire

*Note*: Items rated on a Likert scale -2 = not at all true, -1 = somewhat true; 0 = not true or untrue; 1 = rather true; 2 = very true. *In previous D-MCT studies, this item was included in analyses for module 3.
