# Supplementary material for: Change in negative mental filter is associated with depression reduction in metacognitive training for depression in older adults (MCT-Silver)
Source: Sci Rep. 2024 Jul 25;14:17120. doi: 10.1038/s41598-024-67063-0 (PMC11272923; doi:10.1038/s41598-024-67063-0)
Supplement: Supplementary file 3 — Supplementary Information 3. [file 41598_2024_67063_MOESM3_ESM.docx]

S1. MCT-Silver Modules

| **Module** | **Name** | **Topic** | **Content** |
| --- | --- | --- | --- |
| 1 | Thinking and Reasoning I | Mental filter  Overgeneralization | Exercises illustrate and introduce mental filter and overgeneralization based on everyday situations. Mental filter and overgeneralization are defined and participants practice finding alternative thoughts. Additional strategies to reduce mental filter and overgeneralization are presented (e.g., humor). |
| 2 | Memory | Mood-congruent memory  Cognitive changes due to aging | The module begins with an exercise illustrating the fallibility of human memory, which emphasize that forgetting or remembering events inaccurately is “human.” This principle is then extended to mood-congruent memories and the possible consequences of leaving false memories (particularly for negative situations) unchecked are discussed. How attention and memory can be reduced due to rumination during a depressive episode is illustrated in an engaging exercise. (Normal) changes in thinking and memory that occur in later life are discussed and a series of memory strategies are presented (e.g., having a designated place to hang house keys to prevent forgetting where they were last placed). |
| 3 | Thinking and Reasoning II | “Should” statements  “All or nothing” thinking  Acceptance of negative feelings related to life changes | The module begins by defining and providing examples of “should” statements and “all or nothing thinking”. The pros and cons of holding (overly) high expectations and using “all or nothing” thinking are discussed, and participants are encouraged to consider areas in which they may consider reducing their own (perfectionistic) expectations. The concept of acceptance for negative feelings associated with unchangeable situations (e.g., the loss of a friend) is presented with focus on the consequences of fighting against negative feelings (e.g., rumination) versus allowing them. Participants are encouraged to consider and discuss areas of life in which they would like to practice more acceptance for negative feelings (e.g., family, hobbies). Ways in which older adults can utilize selection, optimization and compensation to find alternative coping strategies are discussed via examples. |
| 4 | Values | (Re-)Identifying values in later life | The importance of defining values in later life is emphasized utilizing a metaphor in which values are likened with a compass while sailing. Examples of values are provided. Differences and similarities between goals and values are clarified. The importance of identifying alternative ways to live out personal values in later life (e.g., due to changes in social roles) is illustrated through engaging exercises. Participants are invited to identify one personal value and to set a small goal of how they may live according to this value. |
| 5 | Thinking and Reasoning III | Magnification and minimization  Depressive attributional style | Participants are asked to think of two things that did and did not go well over the past week. Magnification and minimization are then illustrated when participants remember negative events better than positive events. Identification of “fair” thoughts to counter magnification and minimization is then practiced using everyday examples. The concept of a biased attributional style in depression is defined. Participants gain practice in identifying balanced (versus one-sided) explanations for the occurrence of negative (e.g., car accident) and positive (e.g., receiving a gift) situations. |
| 6 | Behaviors and Strategies | Dysfunctional coping strategies:  Withdrawal,  Rumination,  Thought suppression | Positive metacognitive beliefs regarding the helpfulness of rumination to solve problems are questioned. The difference between rumination and problem-solving is clarified and the futility of attempts to suppress negative thoughts is illustrated with a “white bear” exercise. Mindfulness is offered as an alternative (more helpful) strategy to gain distance from negative thoughts and a breathing exercise is conducted. At the end of the session, the pros and cons of social withdrawal are discussed and an everyday example of negative beliefs that contribute to social withdrawal is discussed. |
| 7 | Thinking and Reasoning IV | Jumping to Conclusions  Mind reading and Fortune-Telling (catastrophizing) | The concepts of mind-reading / fortune-telling and jumping to conclusions are defined and illustrated using everyday examples (e.g., a car parked incorrectly). The pros and cons of attempting to guess others’ thoughts or predict the future are discussed. An engaging exercise in which participants are asked to guess the name of paintings based on the details of the work illustrates how difficult it often is to know what others are thinking. The possible negative consequences of jumping to (incorrect) conclusions and mind reading are discussed. Finally, participants are encouraged to considering multiple sources of information and to practice caution before definitively drawing conclusions. |
| 8 | Self-Esteem | Self-esteem  Changing negative self-perceptions through imagery | Self-worth is defined and the inner and outer characteristics of positive self-worth are identified. An exercise regarding the connection between posture and positive self-esteem is conducted. Negative attitudes toward aging and the consequences of negative comparisons with younger versions of oneself are discussed. To counter predominantly negative attitudes of aging, positive aspects of aging are identified and discussed. Finally, an imagery rescripting exercise aimed at improving negative mental images participants may have of themselves. |
